# Transcriptomics of cytokinin and auxin metabolism and signaling genes during seed maturation in dormant and non-dormant wheat genotypes

**DOI:** 10.1038/s41598-019-40657-9

**Published:** 2019-03-08

**Authors:** Pham Anh Tuan, Yuji Yamasaki, Yuri Kanno, Mitsunori Seo, Belay T. Ayele

**Affiliations:** 1 Department of Plant Science, 222 Agriculture Building, University of Manitoba, Winnipeg, Manitoba, R3T 2N2 Canada; 2 RIKEN Center for Sustainable Resource Science, 1-7-22 Suehiro-cho, Tsurumi-ku, Yokohama, Kanagawa 230-0045 Japan

## Abstract

To gain insights into the roles of cytokinin (CK) and auxin in regulating dormancy during seed maturation in wheat, we examined changes in the levels of CK and indole-3-acetic acid (IAA) and expression patterns of their metabolism and signaling genes in embryonic and endospermic tissues of dormant and non-dormant genotypes. Seed maturation was associated with a decrease in the levels of isopentenyladenine in both tissues mainly via repression of the CK biosynthetic *TaLOG* genes. Differential embryonic *trans*-zeatin content and expression patterns of the CK related genes including *TacZOG*, *TaGLU* and *TaARR12* between maturing seeds of the two genotypes implicate CK in the control of seed dormancy induction and maintenance. Seed maturation induced a decrease of IAA level in both tissues irrespective of genotype, and this appeared to be mediated by repression of specific IAA biosynthesis, transport and IAA-conjugate hydrolysis genes. The differential embryonic IAA content and expression pattern of the IAA biosynthetic gene *TaAO* during the early stage of seed maturation between the two genotypes imply the role of IAA in dormancy induction. It appears from our data that the expression of specific auxin signaling genes including *TaRUB*, *TaAXR* and *TaARF* mediate the role of auxin signaling in dormancy induction and maintenance during seed maturation in wheat.

## Introduction

Wheat (*Triticum aestivum* L.) is one of the most important cereal crops that are cultivated worldwide. Wheat production, however, is negatively affected by a wide range of biotic and abiotic stress factors. Preharvest sprouting (PHS), which is caused by the occurrence of wet and humid conditions prior to harvest, is one of the major factors that significantly reduce wheat yield and quality^1^. The incidence of PHS is tightly associated with the degree of seed dormancy, which is defined as the inability of a viable seed to complete germination under favorable environmental conditions^2^. While the lack of adequate level of dormancy makes the seeds susceptible to PHS, high degree of dormancy negatively affects the rate and uniformity of seed germination and seedling establishment. Therefore, it is necessary to develop wheat cultivars with an optimal level of seed dormancy to mitigate the negative effects of PHS and thereby improve yield and quality. This requires detailed knowledge of the molecular mechanisms underlying the regulation of seed dormancy induction and maintenance during seed maturation.

Seed development is a complex physiological process regulated by intrinsic and extrinsic factors, and in cereal crops such as wheat the process of seed development is characterized by three phases^3,4^. The first phase involves double fertilization that leads to the development of embryo and endosperm, formation of syncytium and cellularization of the endosperm while the second phase involves differentiation of proliferating cells into different specialized cells, endoreduplication and deposition of storage reserves. The third phase of seed development is maturation, which involves shutdown of metabolic activities, programmed cell death, desiccation and induction of dormancy. The type of dormancy acquired by seeds during their maturation on the mother plant is referred to as primary dormancy, and the induction and maintenance of primary dormancy in seeds is regulated by several plant hormones^5,6^. The dynamic balance between two plant hormones, abscisic acid (ABA) and gibberellins (GA), has been considered to be the major regulator of seed dormancy induction and maintenance in both dicot and monocot crop species^5,7,8^. Previous reports have also implicated the participation of other plant hormones such as auxin and cytokinin (CK) in the control of seed dormancy^2,8^. However, the molecular mechanisms underlying the roles of CK and auxin in the regulation of seed dormancy in cereals such as wheat are still poorly understood.

Cytokinin regulates several plant growth and developmental processes via regulating cell proliferation and differentiation^9^. In seeds of some cereal species such as maize and rice, CK is reported to be abundant in the endosperm of developing seeds and this CK enhances cell division in the embryo^10–12^. Consistently, seeds of Arabidopsis mutants defective in CK signaling genes, *Arabidopsis histidine phosphotransfer protein*s (*AHP*s) and *Arabidopsis histidine kinase*s (*AHK*s) are characterized by larger embryos as compared to the wild-type, indicating the role of CK in embryo development^13,14^. The elevated level of CK detected in the endosperm tissue during the early phases of maize and rice seed development has also been reported to be associated with the rapid phase of endospermic cell division^11,12^. The role of CK in the regulation of seed dormancy has been reported to be mediated through modulation of ethylene biosynthesis^10^. For example, CK enhances ethylene synthesis and seed germination in *Striga* species via inducing the expression of ethylene biosynthesis genes, *aminocyclopropane-1-carboxylic acid (ACC)-synthase* (*ACS*)^15^ and *ACC-oxidase* (*ACO*)^16^. Cytokinin has also been proposed to promote seed germination by antagonizing the effects of ABA. For example, the expression of *ABA-insensitive 5* (*ABI5*), a key positive regulator of ABA signaling, has been shown to be repressed by CK signal transducers and transcription repressors, which are designated as type-A Arabidopsis response regulators (ARRs), during seed germination in Arabidopsis^17^. Furthermore, a study in wheat revealed upregulation of a CK biosynthetic *lonely guy* (*LOG*) gene in after-ripened as compared to dormant seeds^18^. Despite these reports, the role of CK in regulating dormancy induction and maintenance during seed maturation in cereals such as wheat remains to be elucidated.

Through its role in promoting cell division and expansion, auxin influences a wide range of plant growth and developmental processes^19,20^. For example, auxin has a basic role in determining embryo structure and size during seed development^21–23^. Given that a high amount of indole-3-acetic acid (IAA), the main naturally occurring auxin, is detected throughout all stages of seed development in cereals such as maize, it has been suggested that auxin plays key roles in regulating seed developmental processes^24–26^. Auxin has also been implicated in the regulation of seed dormancy induction and maintenance^8^. For example, auxin inhibits seed germination in Arabidopsis, and this appears to be associated with its synergistic interaction with ABA in which less amount of ABA is required to inhibit germination in the presence of auxin, indicating that auxin increases seed ABA sensitivity^27^. Auxin is also reported to recruit the auxin response factors (ARFs) to control the expression of *ABA-insensitive 3* (*ABI3*), one of the major regulators of seed dormancy^28^. Furthermore, Arabidopsis seeds over-expressing *miR160*, which represses *ARF10*, exhibit reduced sensitivity to ABA during germination while those over-expressing *miR160*-resistant form of *ARF10* are hypersensitive to ABA^29^. It has been shown recently that the role of auxin in repressing seed germination is mediated through modulation of the GA/ABA ratio^30^. Previous studies have also implicated auxin in regulating germination and dormancy in cereal seeds. For example, treatment of wheat seeds with exogenous IAA or IAA precursors inhibits germination while IAA biosynthesis inhibitors or antagonists of IAA overcomes the germination inhibitory effects of IAA or the precursors^31,32^. Furthermore, higher amount of IAA and enhanced expression of *aldehyde oxidase* (*AO*), an auxin biosynthetic gene, were observed in imbibing dormant than after-ripened wheat seeds^33^. However, little is known about the role of auxin and the associated molecular mechanisms in the regulation of dormancy induction and maintenance during seed maturation in wheat. To gain insights into the roles of CKs and auxin in the regulation of dormancy induction and maintenance during seed maturation in wheat, the present study performed comparative analysis of the expression of CK and auxin metabolism and signaling genes, and CK and IAA levels in the embryo and endosperm tissues of maturing seeds of dormant and non-dormant wheat genotypes.

## Results and Discussion

### Transcriptional regulation of cytokinin metabolism and signaling genes in dormant and non-dormant seeds during maturation

#### Cytokinin metabolism

The first and rate limiting step in the biosynthesis of CK involves the addition of a prenyl group of dimethylallyl diphosphate (DMAPP) onto AMP, ADP or ATP, and this reaction, which is catalyzed by isopentenyl transferase (IPT), leads to the formation of isopentenyladenine (iP)-nucleotides^34^. The iP nucleotides are then converted to *trans*-zeatin (*t*-zeatin) nucleotides by the action of cytochrome P450 monooxygenases (CYP735As)^35^. Cytokinin nucleoside 5′-monophosphate phosphoribohydrolase, also known as lonely guy (LOG), directly activates both iP and *t*-zeatin nucleotides to free-base bioactive CKs including zeatin and iP^36^. The level of bioactive CKs is also regulated via its inactivation by CK oxidase/dehydrogenase (CKX), and conjugation by *cis* zeatin-*O*-glucosyltransferase (cZOG) and reactivation by β-glucosidase (GLU)^34^.

**Figure 1:**
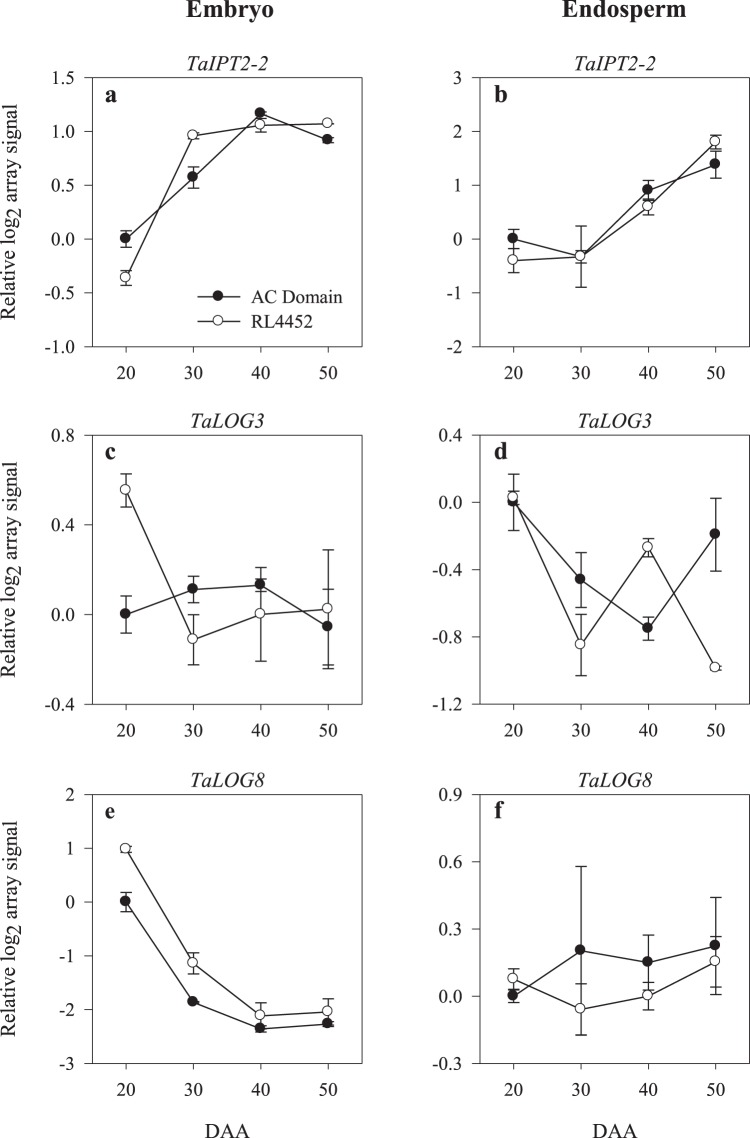
Expression patterns of CK biosynthetic genes (*TaIPT2-2*, *TaLOG3* and *TaLOG8*) in the embryo (**a**,**c**,**e**) and endosperm (**b**,**d**,**f**) tissues of AC Domain and RL4452 during seed maturation. Log_2_ transformed array signal for each gene was expressed relative to that derived from AC Domain sample at 20 DAA, which was set to a value of 0. Asterisks indicate statistically significant differences in expression level between AC Domain and RL4452 samples (≥1-fold on log_2_-scale or ≥2-fold on symmetrical/linear-scale and *P* ≤ 0.05). Log_2_-scale and symmetrical/linear-scale fold changes in expression and the respective *P* values for the embryo and endosperm tissues are presented in Supplementary Tables S1 and S2. *IPT*, *isopentenyltransferase*; *LOG*, *lonely guy*.

**Figure 2:**
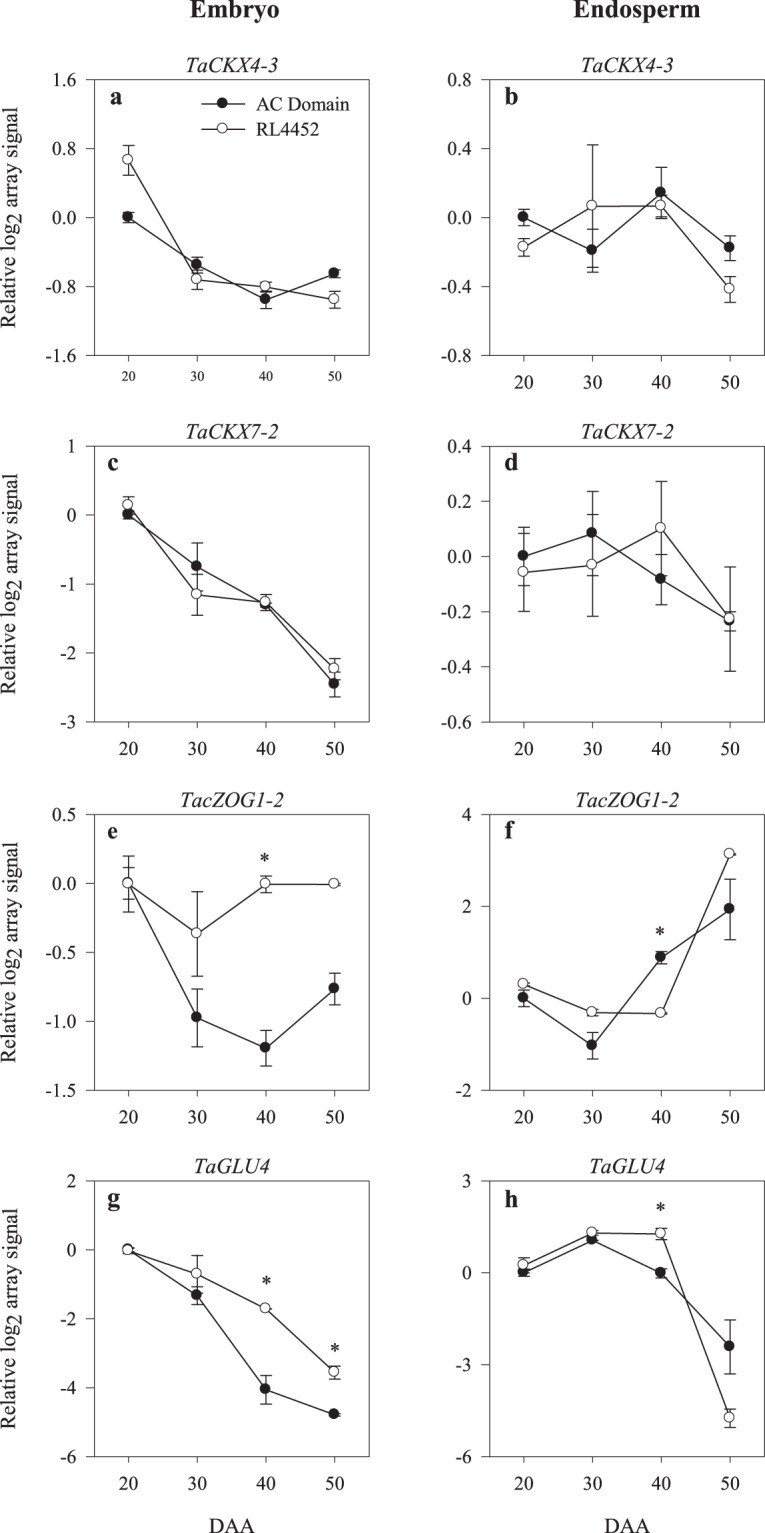
Expression patterns of CK inactivation, conjugation and reactivation genes (*TaCKX4-3*, *TaCKX7-2*, *TacZOG1-2* and *TaGLU4*) in the embryo (**a**–**c**,**e**,**g**) and endosperm (**b**,**d**,**f**,**h**) tissues of AC Domain and RL4452 during seed maturation. Data descriptions are as indicated in Fig. 1. *CKX*, *cytokinin oxidases/dehydrogenase*; *cZOG*, *cis zeatin-O-glucosyltransferase*; *GLU*, *glucosidase*.

**Figure 3:**
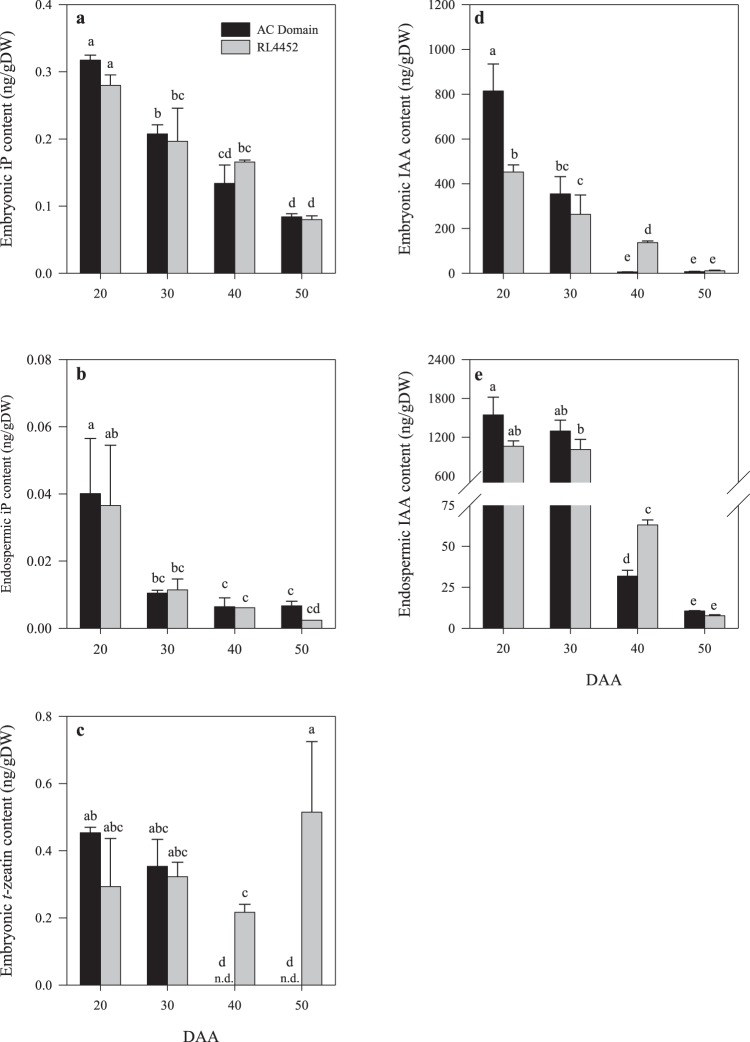
Endogenous isopentenyladenine (iP) and *t*-zeatin, and IAA contents in the embryo (**a**,**c**,**d**) and endosperm (**b**,**e**) tissues of AC Domain and RL4452 during seed maturation. Data are means of three biological replicates ± SE. Different letters denote statistically significant difference in endogenous iP, *t*-zeatin and IAA contents of the embryo and endosperm tissues between genotypes and seed maturation stages at *P* < 0.05. n.d., not detected.

To gain insights into the molecular mechanisms underlying the regulation of CK metabolism and signaling during seed maturation and their subsequent roles in dormancy induction and maintenance, we extracted the expression patterns of CK metabolism and signaling genes from our microarray based transcriptomics analysis of maturing wheat seeds of two genotypes, which are reported to exhibit contrasting seed dormancy phenotypes^37,38^. The microarray data was also validated by RT-qPCR analysis of selected genes, and the microarray and RT-qPCR data exhibited a high degree of positive correlation (*r* ≥ 0.85) for samples derived from both tissues and genotypes (Supplementary Fig. S1). The wheat GeneChip consists of 27 probesets annotated as CK metabolism genes (Supplementary Tables S1 and S2). Seed maturation in both genotypes was associated with induction in the expression level of a probeset annotated as *TaIPT2-2* in both embryo and endosperm tissues with no apparent differential expression between the two genotypes (Fig. 1a,b). Furthermore, the expression levels of probesets representing *TaCKX4-3* and *TaCKX7-2* either declined (in the embryo) or were maintained at almost similar levels (in the endosperm) during seed maturation in both genotypes (Fig. 2a–d). Given that *IPT* encodes an enzyme that catalyzes a rate limiting step in CK biosynthesis and CKX catalyzes CK inactivation^34^, our results might suggest that seed maturation is associated with an increase of embryonic and endospermic CK levels. In contrast, iP level in both embryo and endosperm tissues decreased (over 3-fold) with seed maturation irrespective of genotype (Fig. 3a,b) while that of *t*-zeatin, which is detected only in the embryo tissue of both genotypes, either declined to an undetectable level (for AC Domain) or was maintained at a similar level (for RL4452) (Fig. 3c). The absence of any association between the patterns of *TaIPT2-2* expression, and iP and *t*-zeatin accumulation in both tissues and genotypes might suggest that either *TaIPT2-2* is subjected to post-transcriptional regulation or iP and *t*-zeatin accumulation is transcriptionally regulated by other *IPT* genes that were not represented in wheat GenChip as a previous study reported the presence of at least six *IPT* genes in wheat^39^. Another possibility is that the levels of iP and *t*-zeatin in maturing wheat seeds are regulated by other CK metabolism genes. Consistently, the decline in embryonic and endospermic iP level during seed maturation in both genotypes was associated with decreases in the expression levels of two probesets representing embryonic *TaLOG8* and endospermic *TaLOG3* gene (Fig. 1c–f), which encode a CK activating enzyme (LOG) that directly converts inactive CK nucleotides to bioactive free base form of CK^36^. These results along with the expression patterns of the *TaCKX* genes suggest the minimal role of CK catabolism in regulating the level of CKs in both tissues of maturing wheat seeds.

The declines in the levels of iP in the embryo and endosperm tissues of both genotypes and that of *t*-zeatin in the embryo of AC Domain during seed maturation were also associated with decreases in the expression levels of probesets annotated as *TaGLU4* in the corresponding tissues (Figs 2g,h and 3a–c), which encodes an enzyme that releases bioactive CKs from CK-conjugates through hydroxylation^40^. In agreement with our data, CK levels were found to transiently increase immediately after anthesis but decrease with seed maturation in different cereal species such as maize, rice and barley, suggesting that CKs are mainly involved in regulating cell division during the earlier phase of seed development^25,41^. In contrast to that observed for the CK biosynthetic genes, differential expression of two probesets each representing embryonic *TacZOG1-2* and *TaGLU4* was evident between the two genotypes during mid (40 DAA) and/or late (50 DAA) phases of seed maturation; both probesets showed over 2-fold higher levels of expression in the embryos of RL4452 than AC Domain (Fig. 2e,g). However, *TaGLU4* appeared to exhibit more transcriptional activation than that observed for *TacZOG1-2*. Consistently, the embryo of RL4452 but not that of AC Domain exhibited detectable levels *t*-zeatin during the later phases of seed maturation (Fig. 3c). These results suggest a role for hydrolysis of CK-conjugates in the regulation of embryonic bioactive CK levels and thereby the induction and maintenance of seed dormancy. Previous studies have shown that CK promotes germination through enhancing the expression of ethylene biosynthesis genes, *aminocyclopropane-1-carboxylic acid* (*ACC*)*-synthase* (*ACS*)^15^ and *ACC-oxidase* (*ACO*)^16^. Consistently, the presence of higher level of *t*-zeatin in the embryo of RL4452 at 40 and 50 DAA seeds was associated with enhanced expression level of *TaACO2* (Fig. 3c; Supplementary Fig. S2e; Tables S3 and S4). Furthermore, CK has been reported to antagonize ABA-mediated inhibition of seed germination via repressing the expression of *ABI5*^17^ or enhancing the degradation of ABI5 protein^42^; however, a probeset corresponding to *TaABI5* was upregulated in embryo tissue of maturing RL4452 seeds that exhibited higher level of *t*-zeatin than that observed in the corresponding tissue of AC Domain (Supplementary Fig. S2i). It is therefore likely that regulation of TaABI5 by CK occurs at post-transcriptional level or its transcriptional regulation is mediated by a gene that was not represented in wheat GeneChip.

#### Cytokinin signaling

Cytokinin signaling pathway is mediated by a two-component system in which AHKs serve as CK receptors and AHPs transmit the signal from AHKs to nuclear ARRs^43^. Members of the ARR family can be classified into three groups based on their phylogenetic relationship and domain structure: type-A, type-B and type-C^44^. However, type-A and type-B ARRs are the two main types to be involved in CK signaling^45^. Type-A ARRs consist of a receiver domain and short C-terminal extensions, and act as negative regulators of CK signaling while the type-B ARRs carry MYB-like domains for DNA binding and a glutamine-rich domain for transcriptional activation, and these ARRs act as positive regulators of CK responses. Twenty nine probesets of wheat GeneChip are annotated as genes involved in CK signaling (Supplementary Tables S1 and S2). To identify members of the different groups of ARR, we blast searched the sequences of unigenes corresponding to probesets annotated as *ARR*s (*ARR9* and *ARR12*) against the wheat genome data in Ensembl Plants (http://plants.ensembl.org/Triticum_aestivum/) to obtain their respective full length sequences. Blast searching of the resulting full length sequences against orthologous genes showed that probesets annotated as *TaARR9* and *TaARR12* belong to type-A and type-B ARR groups, respectively.

**Figure 4:**
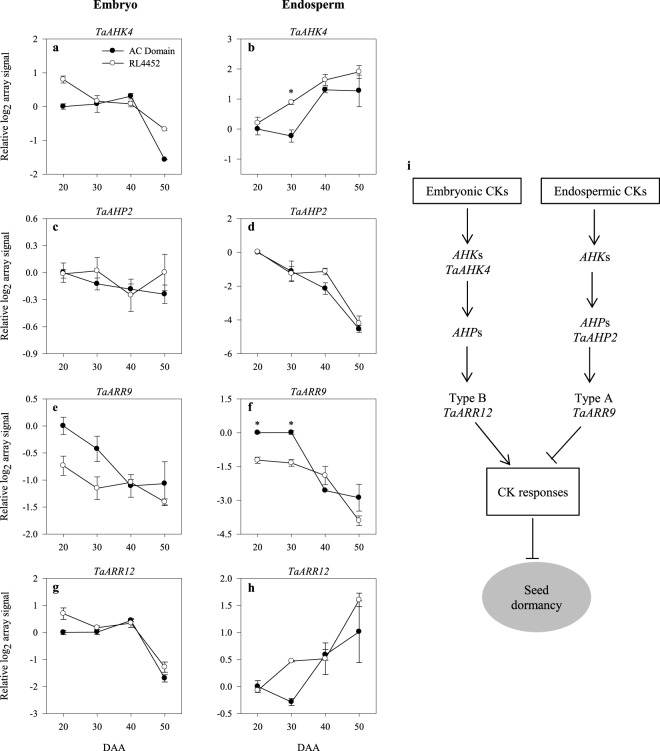
Expression patterns of CK signaling genes (*TaAHK4*, *TaAHP2*, *TaARR9* and *TaARR12*) in the embryo (**a**,**c**,**e**,**g**) and endosperm (**b**,**d**,**f**,**h**) tissues of AC Domain and RL4452 during seed maturation. Data descriptions are as indicated in Fig. 1. Putative model for cytokinin signaling in the embryo and endosperm tissues during seed maturation in wheat and its role in regulating dormancy (**i**). *AHK*, *Arabidopsis histidine kinase*; *AHP*, *Arabidopsis histidine-containing phosphotransmitter*; *ARR*, *Arabidopsis response regulator*; *TaARR12*, type-B ARR (transcriptional activator); *TaARR9*, type-A ARR (transcriptional repressor).

During seed maturation, the expression levels of *TaAHK4*, *TaARR9* and *TaARR12* (Fig. 4a,e,g) exhibited a decrease in the embryonic tissue of both genotypes, and this was associated with a decline in the accumulation of iP and *t*-zeatin (Fig. 3a,c). However, *TaARR12* appeared to exhibit lower level (1.6-fold; *P* = 0.015) of expression in the embryo of 20 DAA AC Domain seeds as compared to that observed in the corresponding RL4452 seeds (Fig. 4g; Supplementary Table S1). Given that TaARR12 belongs to type-B ARR group and acts as positive regulators of CK responses^45^, it is likely that *TaARR12* represses CK response in the embryos of AC Domain and thereby plays a role in the induction of dormancy during seed maturation in wheat. With respect to the endosperm, the expression of a probeset representing *TaAHP2* decreased with seed maturation in both genotypes (Fig. 4d). However, a probeset annotated as *TaAHK4* exhibited lower levels of expression in the endosperm of AC Domain than that observed in RL4452 during the mid phase of seed maturation (30 DAA) (Fig. 4b). Given that AHK serve as CK receptors^43^, our data might suggest increased response to CK and therefore reduced dormancy in RL4452 seeds. The expression levels of endospermic *TaARR9*, on the other hand, was higher in AC Domain than RL4452 seeds during the early to mid phase of seed maturation (20 and 30 DAA) (Fig. 4f). Since *TaARR9* belongs to type-A ARR group that negatively regulate CK signaling^45^, our data suggest decreased CK responses in the endosperm of AC Domain seeds that are characterized by high level of dormancy at maturity. Furthermore, the repression of a probeset corresponding to AHK, the principal receptors of CK^46,47^, in the endosperm of AC Domain might suggest suppression of CK response and thereby induction of dormancy. However, a previous study showed that mutation in *AHK*s results in reduced seed dormancy level and enhanced germination^14^, suggesting the presence of distinct pathways mediating the role of CK in regulating seed dormancy and germination^8^. Based on our data *TaARR9* and *TaARR12* appear to have a tissues specific role in the regulation CK response and thereby dormancy induction and maintenance during seed maturation in wheat as shown in the putative model presented in Fig. 4i.

### Transcriptional regulation of auxin metabolism and signalling genes in dormant and non-dormant seeds during maturation

#### Auxin metabolism

The major bioactive auxin in plants, indole-3-acetic acid (IAA), is synthesized from tryptophan via indole-3-pyruvic acid, and this involves consecutive reactions catalyzed by tryptophan aminotransferase (TAA) and YUCCA (YUC)^48^. IAA synthesis from tryptophan may also take place via indole-3-acetamide in which indole-3-acetamide is converted to IAA by indole-3-acetamide hydrolase (AMI1)^49^. The formation of IAA is also proposed to take place from tryptamine via indole-3-acetaldehyde by the actions of tyrosine decarboxylase (TDC) and AO^49^. Bioactive IAA can be converted to inactive conjugates by glycoside hydrolase 3 (GH3), while several enzymes including IAA-leucine resistant 1 (ILR1), IAA-alanine resistant 3 (IAR3), and IAA-leucine resistant 1-like (ILL) hydrolyse IAA-conjugates to free IAA^50^. Auxin distribution in plants such as during seed development depends on its polar transport between cells, and this is regulated by auxin transporter proteins including PIN-FORMED (PIN) auxin efflux carriers^51^.

**Figure 5:**
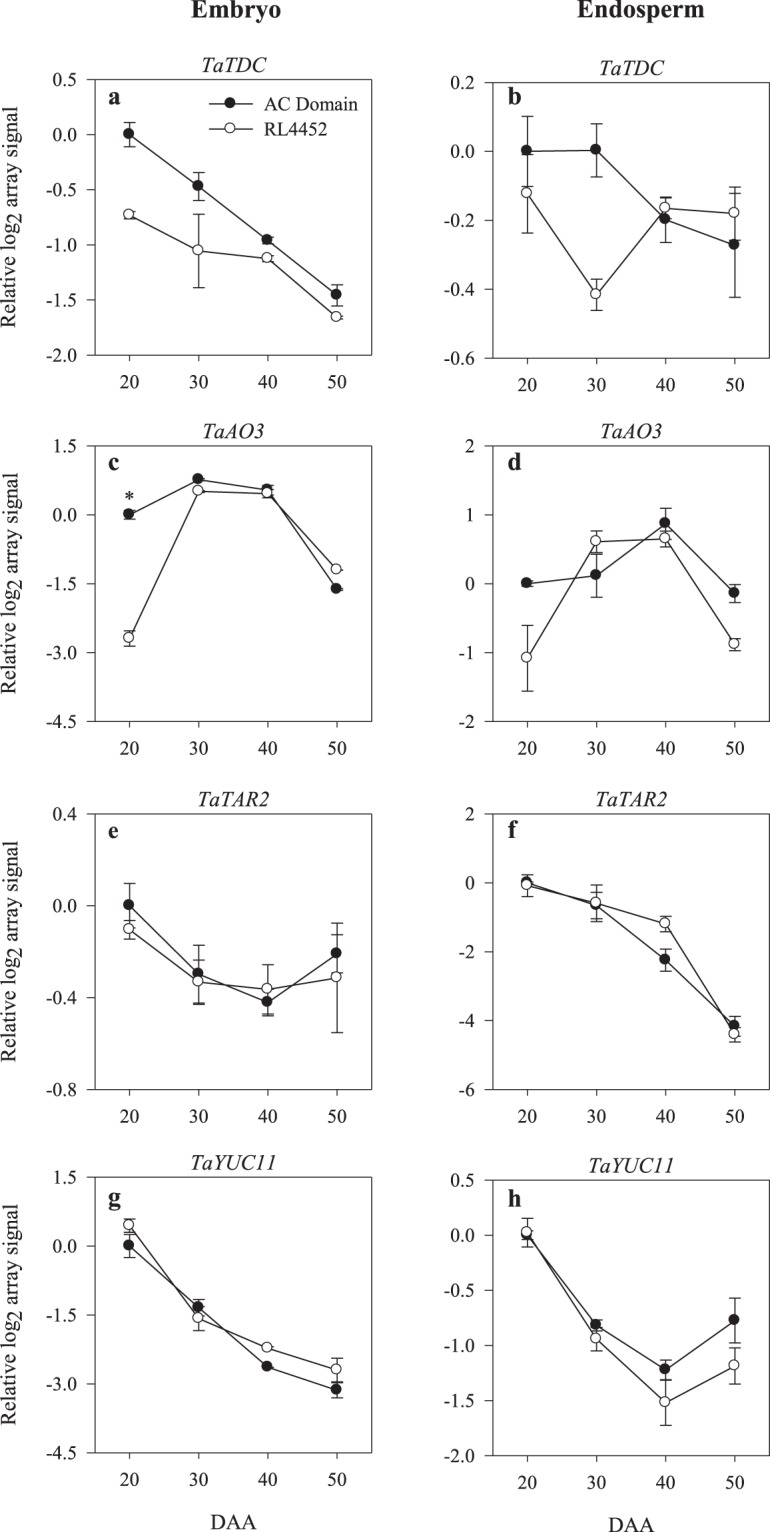
Expression patterns of auxin biosynthetic genes (*TaTDC*, *TaAO3*, *TaTAR2*, and *TaYUC11*) in the embryo (**a**,**c**, **e**,**g**) and endosperm (**b**,**d**,**f**,**h**) tissues of AC Domain and RL4452 during seed maturation. Data descriptions are as indicated in Fig. 1. *TDC*, *tyrosine decarboxylase*; *AO*, *aldehyde oxidase*; *TAR*, *tryptophan aminotransferase related*; *YUC*, *flavin monooxygenases*.

**Figure 6:**
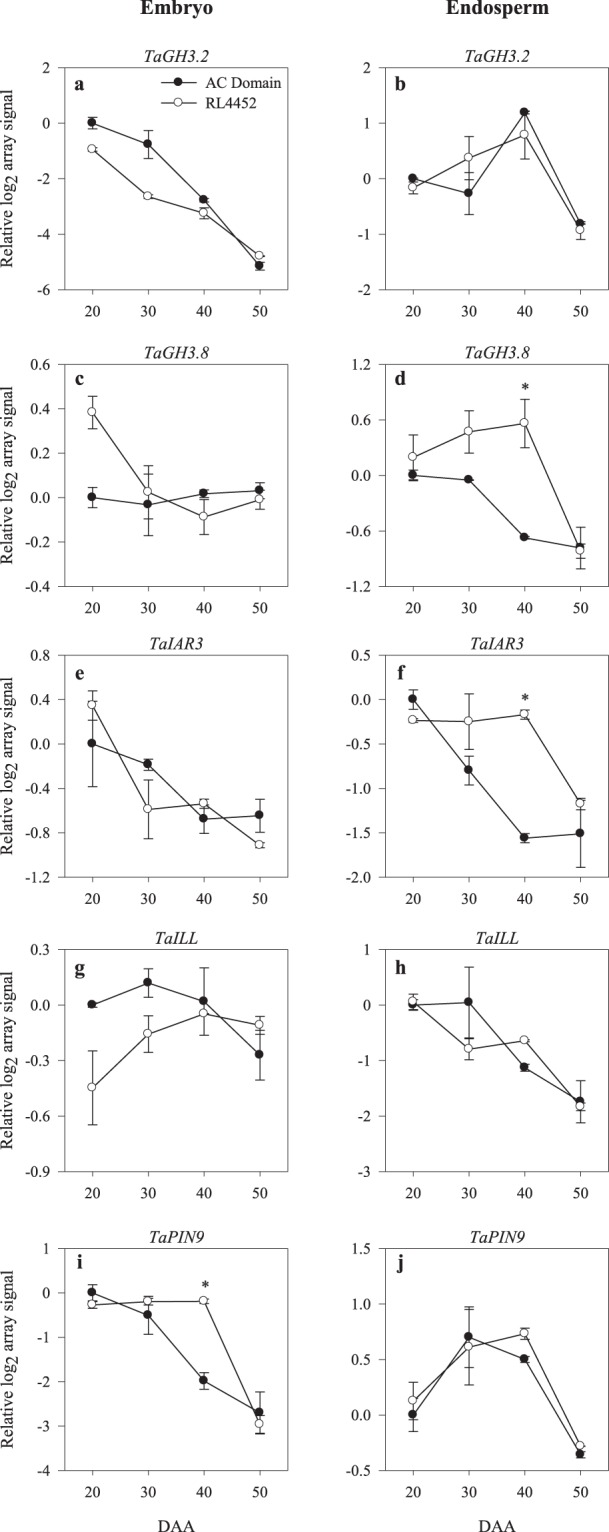
Expression patterns of auxin conjugating (*TaGH3*.*2* and *TaGH3*.*8*), auxin-conjugate hydrolysis (*TaIAR3* and *TaILL*) and auxin transport (*TaPIN9*) genes in the embryo (**a**,**c**,**e**,**g**,**i**) and endosperm (**b**,**d**,**f**,**h**,**j**) tissues of AC Domain and RL4452 during seed maturation. Data descriptions are as indicated in Fig. 1. *GH3*, *Gycoside hydrolase 3*; *IAR3*; *IAA-alanine resistant 3*; *ILL*; *IAA-leucine resistant 1-like*; *PIN*, *PIN-FORMED*.

Thirty six probesets of the wheat GeneChip are annotated as auxin metabolism/transport genes (Supplementary Tables S1 and S2). The expression levels of several probesets representing genes involved in the biosynthesis/transport of bioactive IAA or its release from IAA-conjugates including *TDC*, *AO*, *TAA related* (*TAR*), *YUC*, *IAR3* and *ILL* declined with seed maturation in the embryo (*TaTDC*, *TaAO3*, *TaYUC11*, *TaIAR3*, and *TaPIN9*) and endosperm (*TaAO3*, *TaTAR2*, *TaYUC11*, *TaIAR3* and *TaILL*) tissues of both genotypes (Figs 5 and 6), suggesting a decrease in seed IAA level during seed maturation. In agreement with this, higher level of IAA was detected in both embryonic and endospermic tissues during the early (20 DAA) and mid (30 DAA) phases than the later phases of seed maturation irrespective of genotype (Fig. 3d,e). Similar results have also been reported in maturing seeds of other plant species such as Arabidopsis and maize^52,53^. The decreases in the expression levels of auxin conjugating genes in the embryo (*TaGH3*.*2*) and endosperm (*TaGH3*.*8*) tissues of maturing seeds of both genotypes (Fig. 6a,d) is associated with a decline in IAA level in both tissues, and these results suggest negative feedback regulation of the *GH3* genes due to seed maturation-induced decline in IAA accumulation. The presence of higher level of IAA in the endosperm than in the embryo of both genotypes (Fig. 3d,e) might suggest that the endosperm is a major source of IAA during seed maturation. A previous study has also shown the significance of endospermic auxin not only for endosperm development but also for the formation of seed coat^26,54^.

A probeset annotated as *TaAO3* exhibited a higher level of expression (over 6-fold) in the embryo of AC Domain than that of RL4452 during the early phase of seed maturation (20 DAA) (Fig. 5c), suggesting an increase in IAA synthesis. Consistently, more embryonic IAA (1.8-fold) was detected in AC Domain than that observed in RL4452 during the same phase of seed maturation (Fig. 3d). Given that exogenous IAA has been shown to inhibit seed germination in wheat^31^ and more IAA accumulation occurs during imbibition of dormant than non-dormant wheat seeds^33^, our results might imply the involvement of IAA in the regulation of dormancy induction during seed maturation in wheat. In contrast, higher levels of embryonic IAA, and higher expression levels of endospermic *TaIAR3* and embryonic *TaPIN9* were observed during the mid phase of seed maturation (40 DAA) in RL4452 than that observed in AC Domain (Figs 3d and 6f,i). These results might suggest that the level of dormancy in mature wheat seeds is not dependent on IAA level manifested at maturity. Consistent with this hypothesis, dry mature dormant and after-ripened seeds were reported to exhibit a similar amount of endogenous IAA^33^. However, the observation of enhanced expression of *TaPIN9* in 40 DAA embryo samples of RL4452 that exhibited higher level of *t*-zeatin was not consistent with previous reports that implicated CK as a negative regulator of PIN auxin transporters^55^.

#### Auxin signaling

Auxin binds directly to its receptor designated as transport inhibitor response/auxin signaling F-box protein (TIR1/AFB), which in turn assembles into Skp1-cullin-F-box (SCF) E3 ubiquitin ligase complex, and thereby promotes the interaction between TIR1/AFB and auxin/indole-3-acetic acid (Aux/IAA)^56^. This leads to the degradation of Aux/IAA that inhibits the ARFs, which act as important regulators of the expression of auxin responsive genes. The regulation of SCF E3 ubiquitin ligase is a highly dynamic process involving several proteins including auxin-resistant (AXR) and related to ubiquitin (RUB) that act as positive regulators of auxin responses^57^.

**Figure 7:**
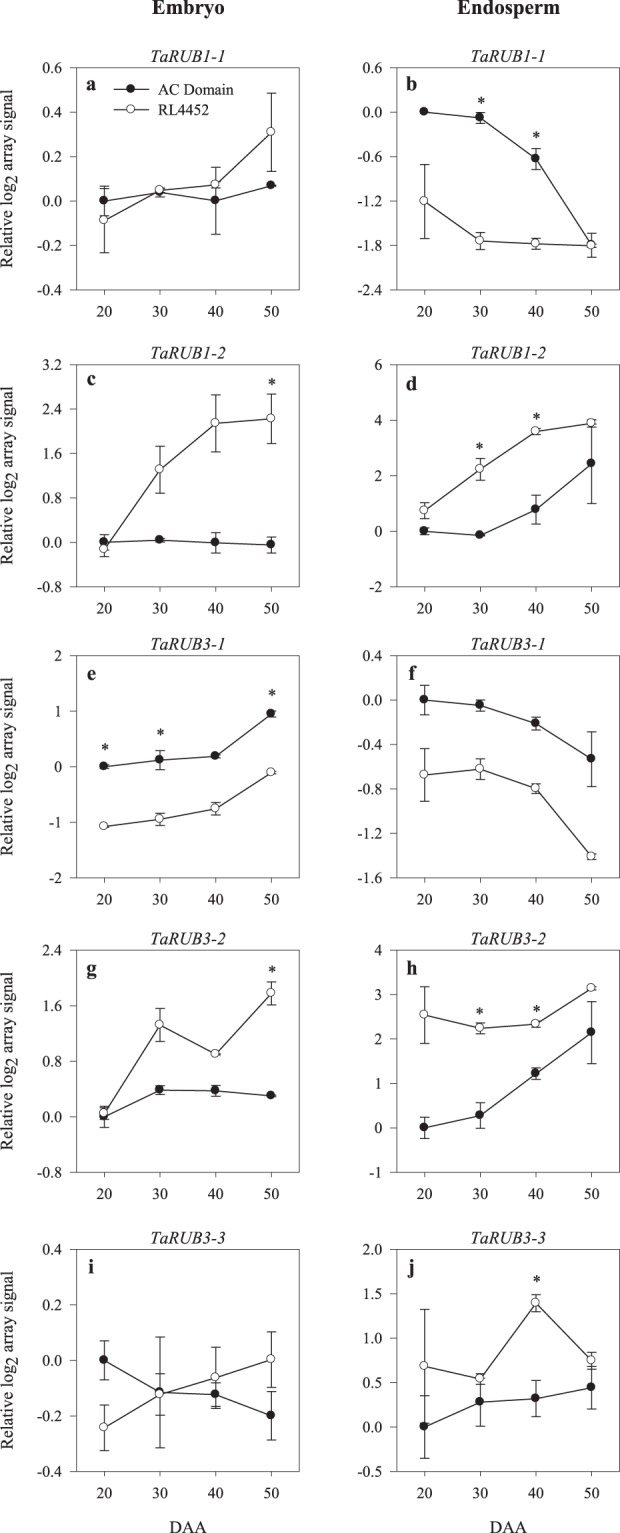
Expression patterns of auxin signaling genes (*TaRUB1-1*, *TaRUB1-2*, *TaRUB3-1*, *TaRUB3-2* and *TaRUB3-3*) in the embryo (**a**,**c**,**e**,**g**,**i**) and endosperm (**b**,**d**,**f**,**h**,**j**) tissues of AC Domain and RL4452 during seed maturation. Data descriptions are as indicated in Fig. 1. *RUB*, *related to ubiquitin*.

**Figure 8:**
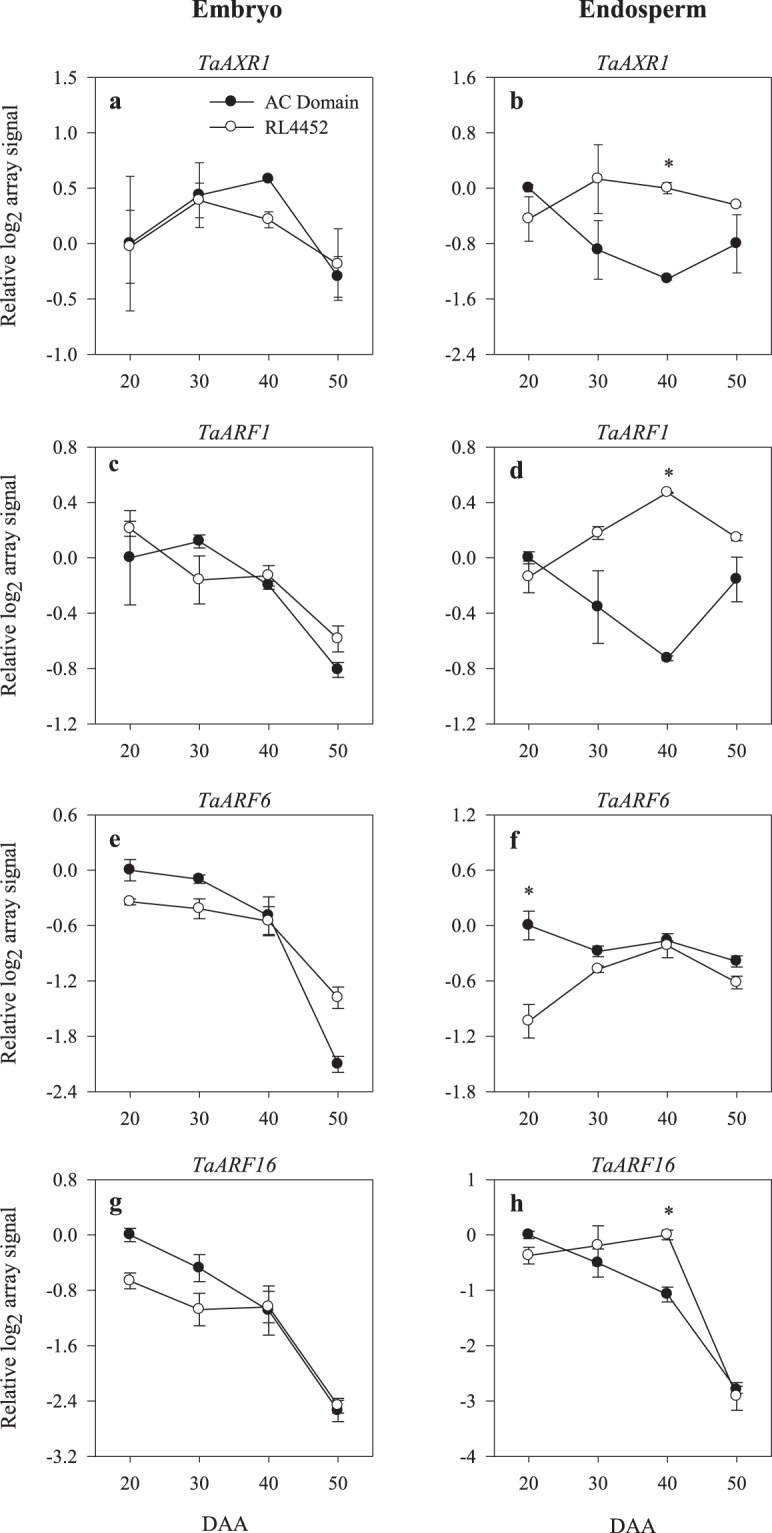
Expression patterns of auxin signaling genes (*TaAXR1* and *TaARF*s) in the embryo (**a**,**c**,**e**,**g**) and endosperm (**b**,**d**,**f**,**h**) tissues of AC Domain and RL4452 during seed maturation. Data descriptions are as indicated in Fig. 1. *AXR*; *auxin-resistant*; *ARF*, *auxin response factor*.

The wheat GeneChip consists of 78 probesets annotated as genes involved in auxin signaling (Supplementary Tables S1 and S2). Our analysis showed that probesets representing *TaRUB1-2* and *TaRUB3-2* exhibited transcriptional repression in the embryo of AC Domain during the late phase of seed maturation (50 DAA) as compared to that observed in RL4452 (Fig. 7c,g). Furthermore, probesets corresponding to *TaRUB1-2*, *TaRUB3-2* and *TaRUB3-3* genes showed transcriptional repression in the endosperm of AC Domain during the mid phase of seed maturation (30 and/or 40 DAA) (Fig. 7d,h,j). Since loss-of-function mutation in *RUB* leads to reduced auxin responses^58^, and a decrease in auxin signaling due to loss of function mutation in one of the *ARF* genes causes increased seed sensitivity to ABA and thereby enhanced dormancy^59^, our data might suggest that specific *RUB* genes are involved in the induction/maintenance of dormancy during seed maturation in wheat. However, a probeset annotated as *TaABI5*, one of the major components of ABA signaling, showed transcriptional repression in both tissues of AC Domain (Supplementary Fig. S2i,j). As discussed above, it is possible that *TaABI5* is regulated post-transcriptionally or transcriptional regulation of ABA signaling is mediated by another *TaABI5* gene not represented in the wheat GeneChip used in this study. The *AXR1* gene of Arabidopsis encodes a subunit of a heterodimeric RUB-activating enzyme, indicating its significance for auxin responses^60,61^. Therefore, the transcriptional repression (over 2-fold) of endospermic *TaAXR1* during the mid phase of seed maturation (40 DAA) in AC Domain relative to that observed in RL4452 seeds (Fig. 8b) might imply that *TaAXR1* is involved in the repression of auxin signaling and thereby induction/maintenance of dormancy in wheat seeds.

Although the auxin response element (AuxRE) is not among the most enriched motifs in maturing wheat seeds^62^, it appears from our results that seed maturation was associated with decreases in the expression levels of probesets annotated as the auxin signaling gene, *ARF*, which functions as transcriptional activator or repressor of auxin response via binding to auxin responsive elements (AuxREs) of its target genes^63–65^, in both embryo and endosperm tissues irrespective of genotype (Fig. 8c,e,g,h). Furthermore, the temporal differential expression pattern of *ARF*s in maturing seed tissues implies the requirement of varied auxin response for the induction of dormancy and completion of seed maturation in wheat. For example, endospermic *TaARF1* exhibited transcriptional repression (over 2-fold) during the mid phase of seed maturation (40 DAA) in AC Domain as compared to that observed in RL4452 (Fig. 8d). Since ARF1 has been reported to function as transcriptional repressor^66^, our data might suggest a role for *TaARF1* in suppressing seed auxin response. In contrast, the observation of transcriptional induction (over 2-fold) of *TaARF6*, which belongs to the ARF transcriptional activator group^66^, in the endosperm of AC Domain during the early phase of seed maturation (20 DAA) relative to that observed in RL4452 (Fig. 8f) might imply increased auxin responses and thereby induction of dormancy during seed maturation. In agreement with this, specific ARFs have been reported to have roles in the regulation of seed dormancy and germination through modulating ABA response. For example, mutation in *ARF2* of Arabidopsis leads to enhanced seed sensitivity to ABA and thereby enhanced dormancy while its overexpression results in reduced ABA sensitivity of seeds and thereby low level of dormancy^59^. The role of auxin in regulating seed dormancy, however, appears to be mediated by distinct auxin signaling pathways since other ARFs such as ARF16 of Arabidopsis have been shown to act as positive regulators of ABI3^28^, which acts as a major component of ABA signaling, and seed dormancy. A probeset annotated as endospermic *TaARF16*, however, exhibited over 2-fold upregulation during the mid phase (30/40 DAA) of seed maturation in RL4452 as compared to that observed in AC Domain (Fig. 8h), and this result might suggest that *TaARF16* is subjected to regulation at post-transcriptional level. Furthermore, no enhanced expression of a probeset annotated as *TaABI3* was observed in the endosperm of AC Domain as compared to that observed in RL4452 (Supplementary Fig. S2h).

In summary, our results provide new insight into the molecular mechanisms underlying the control of CK and auxin metabolism and signaling during seed maturation in wheat and their potential contribution in the regulation of dormancy induction and maintenance.

## Materials and Methods

### Plant materials

Two spring wheat genotypes, AC Domain and RL4452, which exhibit contrasting phenotype in seed dormancy, were used for this study. AC Domain is a registered cultivar in western Canada and is characterized by a high level of seed dormancy at maturity while RL4452 is unregistered breeding line that exhibits a low level of seed dormancy. Seeds of AC Domain and RL4452 were supplied by Dr. Mark Jordan of Agriculture and Agri-Food Canada (AAFC) - Morden Research and Development Center (Morden, Manitoba, Canada). Wheat plants of the two genotypes were grown in a growth chamber at 22 °C/18 °C (day/night) under a 16/8 h photoperiod. Seed maturation in both wheat genotypes was studied from 20 to 50 days after anthesis (DAA), which comprised stages from late phase of storage reserve accumulation to desiccation and divided into early (20 DAA), mid (30 to 40 DAA) and late (50 DAA) phases of seed maturation as described previously^62^. Maturing seed samples were harvested from each genotype at 20, 30, 40, and 50 DAA (~40 seeds per 2–3 spikes per 2–3 plants per replicate for 20, 30 and 40 DAA samples; ~100–120 seeds per 4–6 spikes per 4–6 plants per replicate for 50 DAA samples)^62^. The embryo (including the scutellum) and endosperm (including the aleurone and pericarp) tissues were separated from each seed sample and immediately frozen in liquid nitrogen. Tissues were stored at −80 °C until they were subjected for further analysis.

### RNA isolation

Total RNA extraction from the embryo and endosperm tissues was performed exactly as described before^62^. After verifying their integrity and purity using gel electrophoresis and a spectrophotometer, respectively, the RNA samples were treated with DNase (Ambion, Austin, TX, USA) to remove any contaminant genomic DNA. Prior to the microarray analysis, mRNA was isolated from the total RNA of the endosperm samples using PolyAtract Kit (Promega, Madison, WI, USA). The quality of the total RNA (for the embryo tissues) and mRNA (for the endosperm tissues) samples was further checked using Agilent 2000 Bioanalyzer as described previously^67^.

### Microarray and data analysis

Labeling and hybridization of at least two independent biological replicates of the total RNA (for the embryo samples) and the mRNA (for the endosperm samples) to the Affymetrix GeneChip Wheat Genome Array (Affymetrix, Santa Clara, CA, USA), and subsequent washing, staining and scanning of the arrays were performed as described before^67^. The Affymetrix GeneChip Operating Software was used to convert the data from the 11 probe pairs into a single hybridization intensity, adjust the total signal intensity per chip and determine the number of probesets with “present” detection call. Confirmation of reproducibility of the data obtained from the independent biological replicates was conducted using scatter plot expression analysis as described before^62^. Identification of the probesets expressed in the embryo and endosperm tissue of both genotypes at least at one stage of seed maturation in all replications (*P* < 0.05) was performed with the MAS5 statistical algorithm of FlexArray. The raw intensity data was first normalized with Robust Multi-array Average (RMA) procedure, and then subjected to log_2_ transformation. The microarray dataset supporting the discussion of this article was deposited in the NCBI Gene Expression Omnibus database (GSE83077)^62^.

### Identification of wheat probesets involved in CK, auxin, ethylene and ABA metabolism and signaling pathway

Identification of probesets representing genes involved in CK, auxin, ethylene and ABA metabolism and signaling pathways was performed as described previously^18,33^. Briefly, the CK, auxin, ethylene and ABA metabolism and signaling genes of Arabidopsis, which were obtained from The Arabidopsis Information Resource, were used as queries to search for homologous sequences in the Rice Annotation Project database (http://rapdb.dna.affrc.go.jp/) using a criterion of E-value of <10^−20^. Subsequently, sequences identified from rice and other monocot species were used as queries to search for homologous genes of wheat in the NCBI wheat unigene dataset containing 56,943 unigenes (http://ncbi.nlm.nih.gov/UniGene/UGOrg.cgi?TAXID=4565) as described previously^33^. Identification of the probesets that correspond to the wheat CK, auxin, ethylene and ABA metabolism and signaling gene was performed by blast searching the gene sequences identified against the wheat 61 k microarray platform using the Plant Expression Database (PLEXdb) Blast (http://plexdb.org/modules/PD_general/tools.php) and an E-value of <10^−50^. HarvEST Wheat-Chip (http://harvest.ucr.edu) was used to annotate the candidate probesets (Supplementary Tables S1–S4). Detailed annotation of the the CK, auxin, ethylene and ABA related probesets discussed in this report was performed as described previously^68^. Probesets and their respective genes including the gene names and GenBank IDs are shown in Supplementary Table S5.

### Expression analysis of CK, auxin, ethylene and ABA metabolism and signaling related probesets

In order to identify differentially expressed probesets, the microarray data were analyzed by FlexArray software (http://genomequebec.mcgill.ca/Flex-Array) using analysis of variance (ANOVA) as described before^69^. Fold changes (log_2_ and linear scaled) in expression levels of each target probeset in both embryo and endosperm tissues were compared between seed maturation stages within each genotype (Supplementary Tables S1–S4). In addition, fold changes in expression levels of probesets in the embryo and endosperm tissues at each seed maturation stage were compared between the two genotypes (Supplementary Tables S1–S4). The positive fold change values indicate upregulation in expression, while the negative ones indicate downregulation of expression in each comparison. Probesets with linear ≥2-fold change and *P* ≤ 0.05 are considered to exhibit statistically significant differential expression between seed tissue samples.

### Validation of microarray data using RT-qPCR

To validate the microarray data, expression analysis of selected CK and auxin metabolism and signaling genes was performed in both tissues and genotypes using RT-qPCR and gene specific primers (Supplementary Table S6). To this effect, cDNA samples synthesized from the same total RNA or mRNA samples used for microarray analysis were used as templates for the RT-qPCR assays, which was performed with EvaGreen qPCR Supermix and CFX96 real-time PCR system (Bio-Rad, Hercules, CA, USA) using the thermal conditions described previously^70^. Determination of the relative log_2_ RT-qPCR signal levels of the CK and auxin metabolism and signaling genes was performed using the methods described by Livak and Schmittgen (2001)^71^ after normalization with wheat *β-actin*, which was used as a reference gene.

### Measurement of endogenous CK and IAA contents

The endogenous contents of CKs (iP and *t*-zeatin) and IAA were measured from the embryo and endosperm tissue of three independent biological replicates of maturing seeds. Extraction and purifications of the iP and *t*-zeatin, and IAA from each seed tissue sample was performed as described previously^72^. Quantitative analysis of the endogenous iP and *t*-zeatin, and IAA contents was conducted using liquid chromatography-tandem mass spectrometry (Agilent 6430) as described previously^73^. Statistically significant differences in endogenous CK and IAA contents of the embryo and endosperm between genotypes and seed maturation stages were tested using two-way ANOVA. The means for endogenous CK and IAA contents were compared using Fisher’s least significant difference (LSD) test at *P* < 0.05.

## Supplementary information

Figures S1-S2

Table S1

Table S2

Table S3

Table S4

Table S5

Table S6
