# Supplementary material for: Transcriptomics of cytokinin and auxin metabolism and signaling genes during seed maturation in dormant and non-dormant wheat genotypes
Source: Sci Rep. 2019 Mar 8;9:3983. doi: 10.1038/s41598-019-40657-9 (PMC6408541; doi:10.1038/s41598-019-40657-9)
Supplement: Supplementary file 1 — Figures S1-S2 [file 41598_2019_40657_MOESM1_ESM.pdf]

Supplementary Information

Transcriptomics of cytokinin and auxin metabolism and signaling genes during seed maturation in dormant and non-dormant wheat genotypes

Authors: Pham Anh Tuan[1], Yuji Yamasaki[1], Yuri Kanno[2], Mitsunori Seo[2], Belay T. Ayele[1]

[1]Department of Plant Science

222 Agriculture Building

University of Manitoba

Winnipeg, Manitoba R3T 2N2

Canada

[2]RIKEN Center for Sustainable Resource Science

1-7-22 Suehiro-cho

Tsurumi-ku, Yokohama

Kanagawa 230-0045

Japan

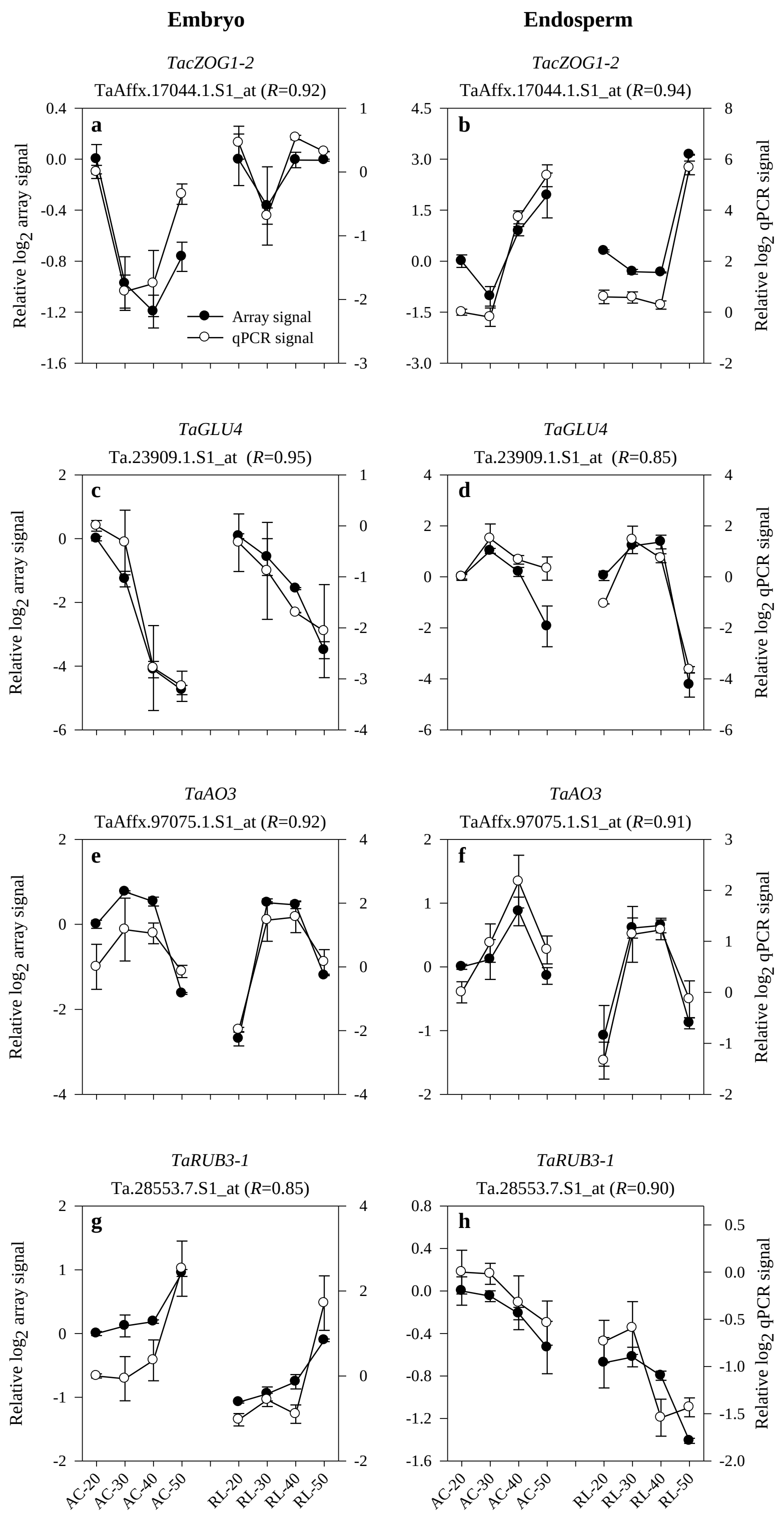

Supplementary Figure S1. Comparison of the expression patterns from microarray and RT-qPCR analysis for selected cytokinin and auxin metabolism and signaling genes in the embryo (a, c, e, g) and endosperm (b, d, f, h) tissues of the two genotypes. Curves with solid circles represents

relative $\log_2$ array signal (left y-axis) while curves with open circles represents relative $\log_2$ RT-qPCR signal (right y-axis). The $\log_2$ array and qPCR signals for each probeset were expressed relative to the respective signals derived from AC Domain sample at 20 days after anthesis (DAA), which was set to a value of 0. AC-20, AC-30, AC-40, and AC-50 indicate AC Domain samples at 20, 30, 40, and 50 DAA; RL-20, RL-30, RL-40, and RL-50 indicate RL4452 samples at 20, 30, 40, and 50 DAA. *cZOG*, *cis zeatin-O-glucosyltransferase*; *GLU, glucosidase*; *AO, aldehyde oxidase*; *RUB*, *related to ubiquitin*.

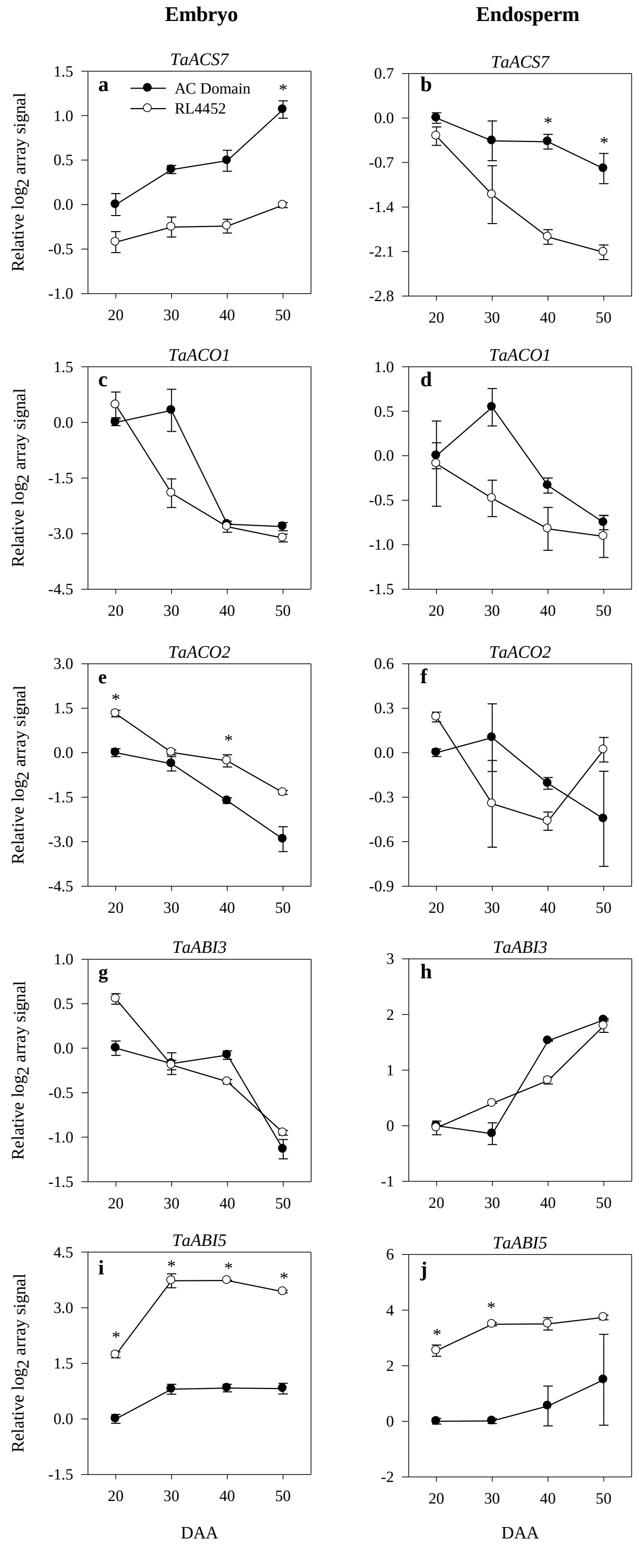

Supplementary Figure S2. Expression patterns of genes related to ethylene biosynthesis (*TaACS7*, *TaACO1* and *TaACO2*) and abscisic acid signaling (*TaABI3* and *TaABI5*) in the embryo (a, c, e, g, i) and endosperm (b, d, f, h, j) tissues of AC Domain and RL4452 during seed maturation. Log_2

transformed array signal for each gene was expressed relative to that derived from AC Domain sample at 20 DAA, which was set to a value of 0. Asterisks indicates statistically significant difference in expression level between AC Domain and RL4452 samples (≥ 1-fold on log_2-scale or ≥ 2-fold on symmetrical/linear-scale and $P \leq 0.05$). Log_2-scale and symmetrical/linear-scale fold changes in expression and the respective *P* values for the embryo and endosperm tissues are presented in Supplementary Table S3 and S4 online. *ACS*, *1-aminocyclopropane-1-carboxylic acid (ACC) synthase*; *ACO*, *ACC oxidase*; *ABI*, *abscisic acid-insensitive*.
